# Inetetamab in combination with rapamycin and chemotherapy for trastuzumab‐treated metastatic human epidermal growth factor receptor 2‐positive breast cancer with abnormal activation of PI3K/Akt/mTOR pathway

**DOI:** 10.1002/cnr2.1864

**Published:** 2023-07-28

**Authors:** Aijuan Wang, Chenghui Li, Qi'an Jiang, Shu Jiang

**Affiliations:** ^1^ Department of Oncology Anqing Municipal Hospital Anqing Anhui China

**Keywords:** anti‐HER‐2 therapy, breast cancer, human epidermal growth factor receptor 2

## Abstract

**Background:**

Human epidermal growth factor receptor 2 (HER2) overexpression is an independent prognostic factor of poor prognosis and a predictor of efficacy of anti‐HER2 therapy. A limited number of patients can receive standard second‐line therapy (DS‐8201 or T‐DM1) for metastatic HER2‐positive in some parts of the world, including China, due to many factors, such as cost–benefit ratios.

**Case:**

A 51‐year‐old premenopausal woman was diagnosed with HER2‐positive breast cancer. The pathological stage was ypT3N2M0 and stage IIIA. Trastuzumab targeted therapy combined with goserelin depot was started along with letrozole endocrine therapy. After eight courses of treatment, magnetic resonance imaging (MRI) examination revealed new multiple metastases in the liver, and progression disease (PD) was evaluated. Due to abnormal activation of the phosphatidylinositol 3‐kinase/protein kinase B/mammalian target of rapamycin (PI3K/AKT/mTOR) pathway in the patient, treatment was changed to the mammalian target of rapamycin (mTOR) inhibitor combined with the anti‐HER‐2 agents inetetamab and paclitaxel, while partial response (PR) was evaluated after 6 cycles of treatment. As the patient was hormone receptor (HR) positive, treatment was changed to the inetetamab + rapamycin + exemestane regimen. The lesion continued to shrink and PR was evaluated for 8 cycles. The original regimen was continued, PR was evaluated after 12 courses of treatment. The abdominal MRI performed showed an increase in the volume of intrahepatic multiple metastatic tumor lesion. Efficacy was used to assess for PD and the progression‐free survival (PFS) was 317 days.

**Conclusion:**

A phosphatidylinositol‐4, 5‐bisphosphate 3‐kinase catalytic subunit alpha (PIK3CA) mutation in trastuzumab‐treated metastatic HER2‐positive breast cancer female had a long PFS by treating with the mammalian target of rapamycin inhibitor in combination with the anti‐HER‐2 agent inetetamab.

## 1 INTRODUCTION

Breast cancer is one of the most frequently diagnosed life‐threatening malignancies in women both in China and abroad.^1^ Human epidermal growth factor receptor 2 (HER2) is a transmembrane protein with receptor tyrosine kinase activity and a member of the epidermal growth factor receptor family, overexpressed in 15%–20% of patients with invasive breast cancers, contributing to poor prognosis for patients.^2^ Currently, anti‐HER2 therapy has been extensively used in clinical practice and trastuzumab had become the standard postoperative targeted therapy for HER2‐positive breast cancer, however, the emergence of drug resistance has made it challenging for patients to benefit from trastuzumab in the long term.^3^ Mutations in the phosphatidylinositol‐4, 5‐bisphosphate 3‐kinase catalytic subunit alpha (PIK3CA) gene are currently one of the known mechanisms of resistance to anti‐HER‐2 therapy and are associated with a poorer prognosis.^4^ Inetetamab is a trastuzumab analogue that has the exact F(ab')2 of trastuzumab fusing into a different IgG1 Fc allotype, with two amino acid variants that differ from trastuzumab, which could be used for treatment after trastuzumab resistance.^5^

This study reported a case of PIK3CA mutation in HER‐2 positive breast cancer patients whose phosphatidylinositol 3‐kinase/protein kinase B/mammalian target of rapamycin (PI3K/AKT/mTOR) pathway was activated abnormally after the primary resistance to trastuzumab, treated with the mammalian target of rapamycin (mTOR) inhibitor in combination with the anti‐HER‐2 agent inetetamab and the second‐line nab‐paclitaxel, the progression‐free survival (PFS) was 317 days and tolerable adverse effects. This study is designed to evaluate the efficacy and safety of inetetamab combined with rapamycin and chemotherapy regimens and to explore new treatment options for trastuzumab‐resistant disease with the goal of improving prognosis.

## 2 CASE PRESENTATION

### 2.1 Chief complain

On May 5, 2020, a 51‐year‐old non‐menopausal woman came to Anqing Municipal Hospital for examination due to a mass in the left breast that has developed for more than 2 months.

### 2.2 Present patient history

The patient had a history of type 2 diabetes mellitus for 10 years and received insulin hypoglycemic treatment with stable blood glucose control, while a history of hypertension for more than 8 years and amlodipine hypoglycemic treatment with stable blood pressure control. The patient denied other specific medical histories.

### 2.3 Examination

The mammography showed hypoechoic masses in the left breast (Breast Imaging‐Reporting and Data System [BI‐RADS] category 5), hypoechoic masses in the left axilla (possible metastasis), echogenic bilateral breast nodules (BI‐RADS category 2), and multiple ductal dilatation effusions in both breasts. Results of the mammographic examination indicated diffuse glandular denseness with mass formation in the left breast, possibly malignant (likely breast cancer, inflammatory lesion with abscess formation to be excluded). Multiple lymph nodes were found in the left axilla. Enhanced computed tomography (CT) scans of the chest and abdomen showed the possibility of left breast cancer with left axillary lymph node metastasis. A tiny cyst was detected in the right infrarenal region. Head magnetic resonance imaging (MRI) showed (1) point‐like abnormal signals next to the posterior horn of the left lateral ventricle, suggesting cerebral ischemia, and (2) cavum septum pellucidum. No obvious abnormality was found by Skeletal ECT.

### 2.4 Diagnosis

Results of the fine needle aspiration of left breast masses performed on May 8, 2020, demonstrated invasive ductal carcinoma of the left breast with visible intraventricular cancer thrombus. Meanwhile, the immunohistochemistry results were as follows: estrogen receptor (ER) (~90%+, strong), progesterone receptor (PR) (~90%+, strong), HER‐2 (3+), and Ki‐67 (~70%+).

### 2.5 Treatment

The patient refused to use targeted therapy due to financial reasons and was administered with six courses of chemotherapy of the TTC regimen (docetaxel 75 mg/kg d1 + pirubicin 50 mg/kg d1 + cyclophosphamide 500 mg/kg d1 q3w). Chemotherapy was initiated on May 13, 2020, and completed on August 30, 2020. The patient experienced adverse effects but was well tolerated.

**FIGURE 1:**
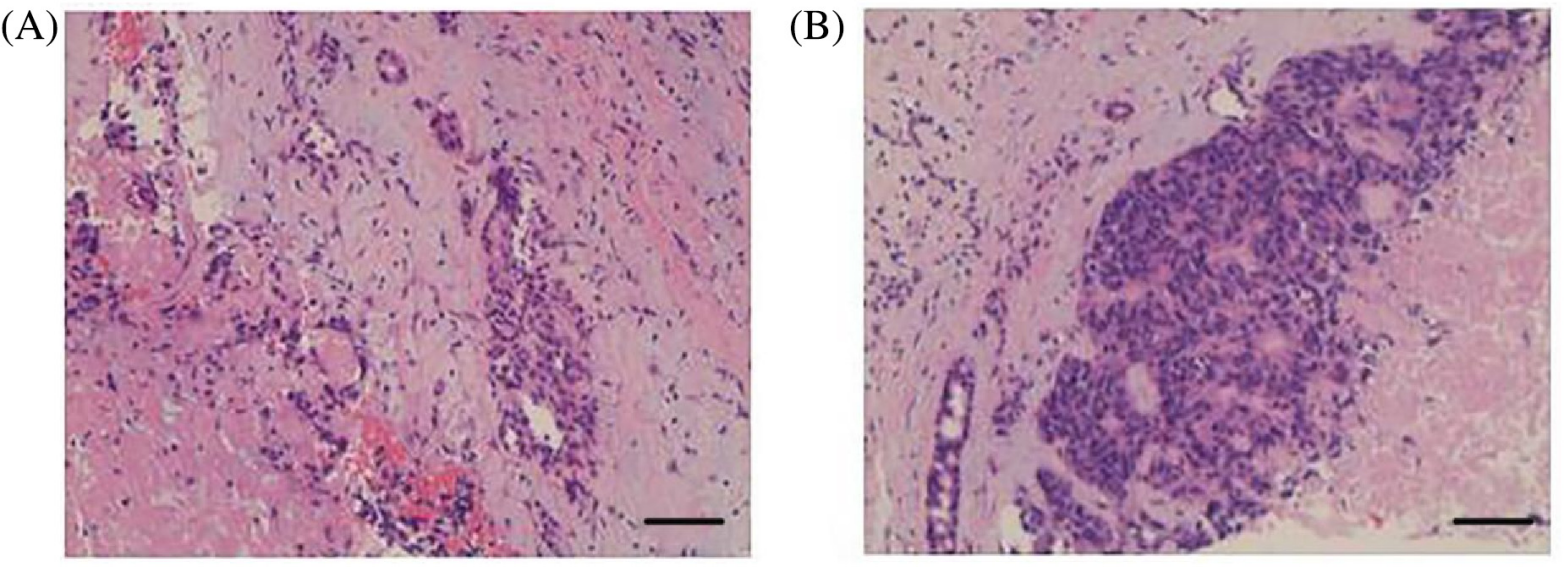
Immunohistochemical analyses. The immunohistochemistry results after modified radical surgery showing CK8 positive; CK5/6: negative for invasive carcinoma; ER: moderate to strong, ~75%+; PR: moderate, ~30%+; HER‐2 score: 3+; and Ki‐67 score: ~15%+. A and B represent images in different fields of view of immunostaining. Black bars: 250 μm (×40).

On September 24, 2020, modified radical surgery for left breast cancer was performed and postoperative pathology showed invasive ductal carcinoma, grade II–III, partly high‐grade ductal carcinoma in situ with necrosis. The mass measured 6 × 4 × 3 cm, and intraventricular cancer thrombus was visible. No carcinoma was noted in the nipple, skin, or basal area. Cancer metastasis was observed in the axillary lymph nodes (4/26). The immunohistochemistry results were as follows: CK8 positive; CK5/6: negative for invasive carcinoma; ER: moderate to strong, ~75%+; PR: moderate, ~30%+; HER‐2 score: 3+; E‐cadherin positive; P120: positive membrane expression; and Ki‐67 score: ~15% + (Figure 1). The pathologic staging was ypT3N2M0, stage IIIA, according to the AJCC staging system eighth edition.

From November 9 to December 11, 2020, postoperative adjuvant radiotherapy for left breast cancer was performed (PTV: 5000 cGy/25f/qd). For economic reasons, the patient declined further undergo dual‐target therapy and was administered with trastuzumab targeted therapy combined with goserelin depot plus letrozole endocrine therapy. The initial trastuzumab dose was 8 mg/kg and followed by a maintenance dose of 6 mg/kg every 3 weeks thereafter. The last targeted therapy was administered on April 13, 2021.

**FIGURE 2:**
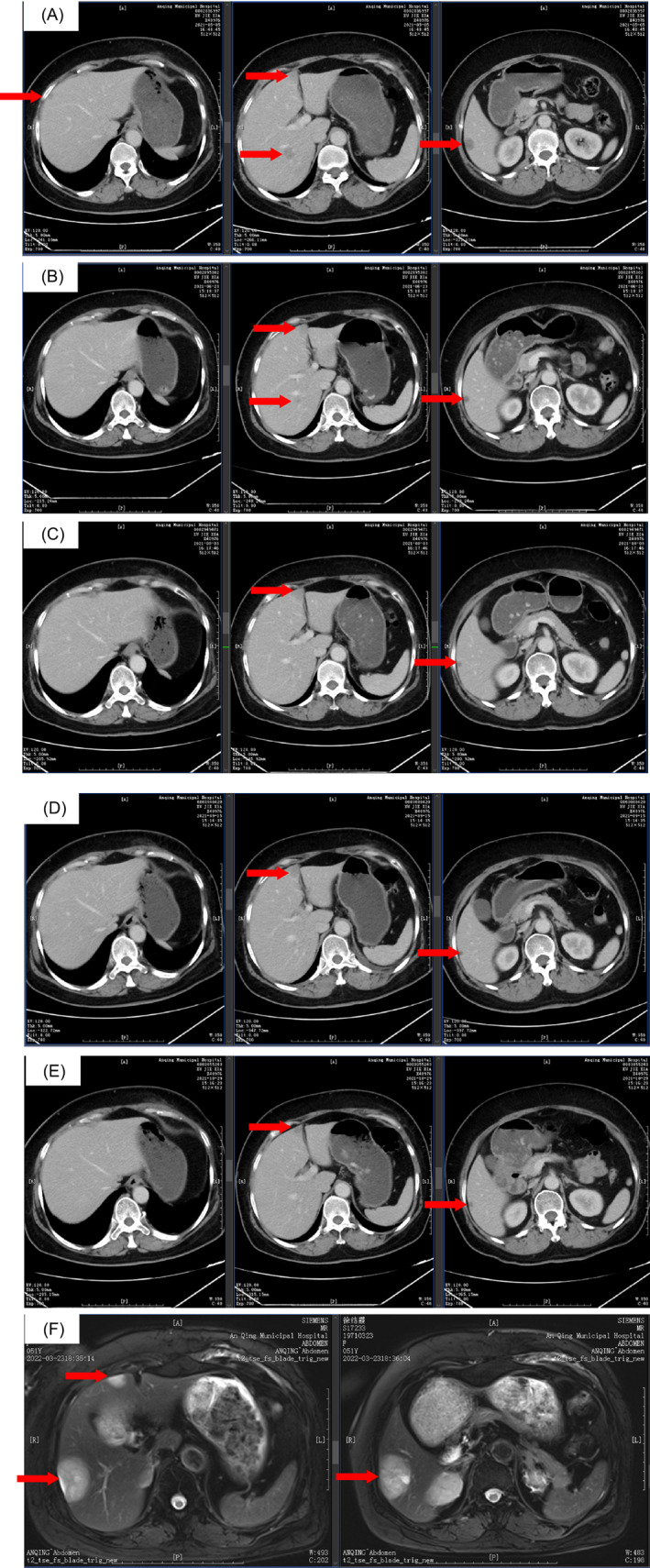
Abdominal CT scan images. (A) Liver metastases prior to the study enrollment (arrow, 2021.05.05 CT). The results revealed low‐density lesions in the right lobe of the liver, with the larger foci about 19 mm in diameter. Cancer metastasis was suspected. (B–D) The abdominal enhanced CT examination at the second, fourth, and sixth cycles of treatment revealed that the tumor continued to shrink (from 12 to 6 mm) and some liver metastases completely disappeared (B: 2021.06.23; C: 2021.08.03; D: 2021.09.15). (E) As the patient was HR positive, the treatment regimen of “Inetetamab + rapamycin + paclitaxel” was changed to “inetetamab + rapamycin + exemestane” for 8 cycles and liver metastases lesion was shrunk to 3 mm (arrow, 2021.10.29 CT). (F) The abdominal MRI showed an increase in the volume of intrahepatic multiple metastatic tumor lesions, with the largest lesion size about 0.5 cm × 3.3 cm, and an abnormal signal in the right adrenal gland (arrows, 2022.03.23 MRI). [Correction added on 5 September 2023, after first online publication: In Figure 2, the positioning of the arrows has been corrected in this version.]

On May 4, 2021, the review of the chest and abdomen enhanced CT results revealed several small calcifications in the right breast gland. Low‐density lesions were observed in the right lobe of the liver, cancer metastasis was suspected a few fibrous foci were found in both lungs. Nodular foci were found in the right adrenal gland and accessory spleen was also noted (Figure 2A). The laboratory test showed an alpha fetoprotein (AFP) 2.5 ng/mL, carcinoembryonic antigen (CEA) 2.9 ng/mL, carbohydrate antigen 125 (CA) 15.4 U/mL, CA19‐9 23.3 U/mL, CA15‐3 6.0 U/mL, CA724 2.2 IU/mL and ferritin (FERR) 60.1 μg/L. On May 12, 2021, the liver MRI examination yielded the following results: (1) Multiple abnormal signals were found in the liver; combined with the patient's medical history, the possibility of metastases was considered. (2) Right adrenal gland occupancy was noted, thus suggesting adenoma. New multiple metastases appeared in the liver, and the disease progression. Letrozole was discontinued.

On May 10, 2021, the patient was enrolled in a clinical trial named “Inetetamab Plus Rapamycin and Chemotherapy for HER2‐positive Metastatic Breast Cancer With Abnormal Activation of PAM Pathway” and the clinical trial ID is NCT04736589, which evaluated the efficacy and safety of inetetamab combined with rapamycin and chemotherapy in patients with trastuzumab‐treated metastatic HER2‐positive breast cancer with abnormal activation of the PI3K/AKT/MTOR pathway. On May 12, 2021, the patient was detected with PIK3CA gene mutation in locus 1047R by digital PCR method and met the inclusion criteria. The patient's random number was 17 001, Inetetamab (an initial dose of 8 mg/kg Q3W, followed by a maintenance dose of 6 mg/kg every 3 weeks) + paclitaxel (albumin‐bound) (260 mg/m^2^ Q3W × 6) + rapamycin (2 mg oral QD) was administered in 6 cycles starting on May 13, 2021. The patient developed grade 2 diarrhea after the first cycle of treatment and was administered with montelukast, which improved the severity of diarrhea, and diarrhea disappeared after 2 cycles of treatment. Results of repeat chest and abdominal enhanced CT examination at the second, fourth, and sixth cycles of treatment reveal that tumor continued to shrink and some liver metastases completely disappeared (Figure 2B–D), indicating a PR effect.

As the patient was hormone receptor (HR) positive, the inetetamab + rapamycin + exemestane regimen was administered on September 18, 2021, for 8 cycles. The lesion continued to shrink after 8 cycles of treatment, and PR was evaluated (Figure 2E). The patient received six more cycles of the original regimen on October 31, 2021, November 23, 2021, December 19, 2021, January 10, 2022, February 8, 2022, and February 26, 2022, for 10 courses; PR was evaluated after 12 courses of treatment. The blood analysis performed on March 23, 2022 revealed the following: AFP of 3.4 ng/mL, CEA of 8.4 ng/mL, CA125 of 31.9 U/mL, CA19‐9 of 44.8 U/mL, CA15‐3 of 7.3 U/mL, CA724 of 0.7 IU/mL and FERR of 91.9 μg/L. The abdominal MRI showed (1) an increase in the volume of intrahepatic multiple metastatic tumor lesions and (2) an abnormal signal in the right adrenal gland, roughly similar to the previous one (Figure 2E). The CT scan demonstrated postoperative changes in the left breast, calcifications in the right breast, and fibrous stripes in the middle lobe of the right and upper lobe of the left lung. Efficacy was used to assess for PD. The patient eventually withdrew from the clinical trial.

## 3 RESULTS

**FIGURE 3:**
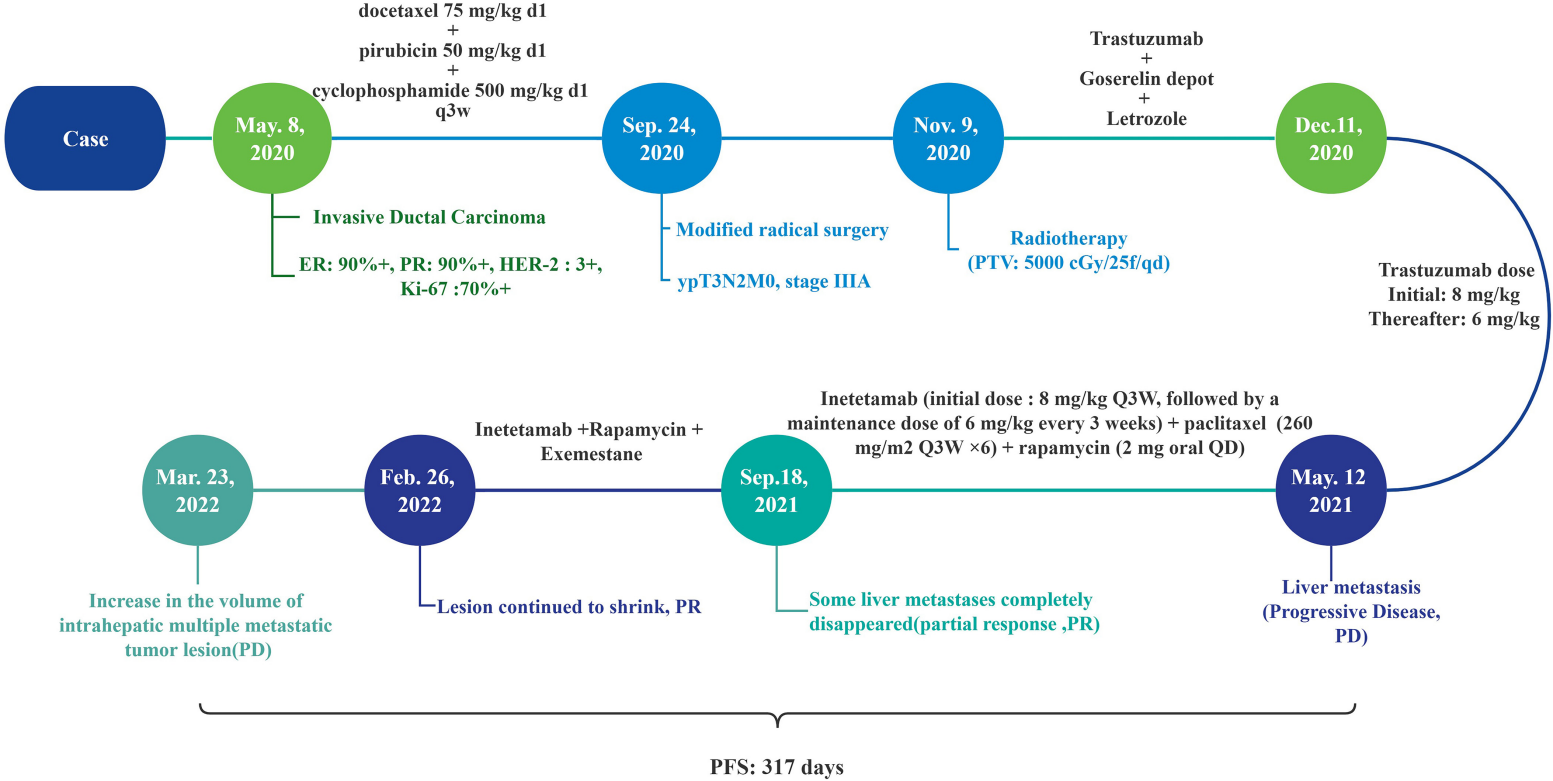
The timeline of therapy and disease status of patient with HER2‐Prositive breast cancer.

The patient who progress to liver metastasis after trastuzumab treatment and had abnormal activation of the PI3K/Akt/mTOR pathway treated with inetetamab combined with rapamycin and chemotherapy achieved 317 days of PFS and tolerable adverse effects. A timeline of the patient's treatment strategy and response are depicted in Figure 3.

Patients showed good tolerability and adherence to the treatment, no severe adverse reactions (except for diarrhea) and unanticipated events have been reported. The patients were followed up by phone call consultation every 2 weeks until withdrew from the clinical trial.

## 4 DISCUSSION

Breast cancer is a type of malignant tumor with the highest morbidity and mortality in women.^6^ HER‐2 overexpression is present in 15%–20% of women with breast cancer and is associated with poor prognosis.^7^ Anti‐HER‐2 therapy has dramatically improved the prognosis of HER‐2‐positive breast cancer patients with a median survival of nearly 5 years and low distant recurrence rate.^8^, ^9^, ^10^ While targeted therapy against HER2 is an effective first‐line treatment in HER2‐positive breast cancer,^4^ acquired resistance remains a clinical challenge.

The patient, in this case, reported experiencing PD within 6 months after receiving adjuvant targeted therapy for postoperative breast cancer with new multiple liver metastases. This finding is consistent with that of Pierobon et al.,^11^ which indicated that PIK3CA mutation was found in 62.5% of patients with liver metastasis, while the rate of PIK3CA mutation was only 5.5% in patients with non‐hepatic lesions, demonstrating that breast cancer patients carrying PIK3CA mutation was significantly greater among liver metastases compared with the other metastatic sites. In conjunction with the medical history, the patient was considered to be resistant to trastuzumab and endocrine treatment. Although HER2‐targeting antibody trastuzumab confers a substantial benefit for patients, acquired resistance remains has become a key challenge in clinical practice. Chinese guidelines also recommend pyrrolitinib + capecitabine as treatment for primary trastuzumab resistance, but both pyrrolitinib and capecitabine are associated with high incidence of grade 3 diarrhea and poor patient compliance. Thus, trastuzumab resistance is an important clinical problem and management for patients with primary trastuzumab resistance is still an unmet clinical need.

PIK3CA mutation is one of the common mechanisms of HER‐2 resistance, with a mutation rate of 12%–39% in HER‐2‐positive breast cancer.^12^ Loibl et al. conducted a meta‐analysis that involving 967 patients with HER2‐positive breast cancer and found that patients with *PIK3CA* mutations treated with neoadjuvant chemotherapy combined with anti‐HER2 therapy (even with a dual‐targeted regimen) had significantly lower rates of pathologic complete remission than those with wild‐type PIK3CA (16.2% vs. 29.6%, *p* < .001).^13^

In the correlation analysis of the CLEOPATRA study, 32% of patients with metastatic HER‐2‐positive breast cancer had a PIK3CA mutation and the PFS in patients with PIK3CA mutation of targeted therapy was significantly lower than those with wild‐type PIK3CA.^14^, ^15^ Ioannis et al.^16^ had also found HER2 cell line HCC1954 carry a strong gain‐of‐function PIK3CA mutation exhibited sustained MTOR signaling. PIK3CA mutations are associated with activation of the PI3K/AKT/mTOR pathway, leading to abnormal activation of this pathway and in turn stimulates tumor proliferation, metastasis, and invasion. Accumulating evidence suggests that PIK3CA mutations are associated with a poorer prognosis and are more likely to lead to drug resistance.^17^

Preclinical studies have implicated that the combination of PI3K/AKT/mTOR pathway inhibitors can enhance the antiproliferative activity of anti‐HER‐2 therapy and overcome resistance to anti‐HER‐2 therapy.^18^, ^19^, ^20^, ^21^ The BOLERO‐3 study showed that everolimus (mTOR inhibitors) in combination with trastuzumab and vincristine significantly prolonged the PFS of HER2‐positive, trastuzumab‐resistant patients who previously received paclitaxel‐based chemotherapy (7.00 months vs. 5.78 months, *p* = .0067).^22^ Meanwhile, PIK3CA gene mutations were associated with endocrine resistance. The BELERO‐2 study showed that for patients with breast cancer who experienced relapse or PD after NSAID treatment, the PFS was significantly prolonged in the everolimus combined with exemestane group compared with that in the placebo combined with exemestane group (7.8 months vs. 3.2 months, *p* < .01).^23^ However, the clinical application of everolimus is limited due to its many adverse reactions, poor tolerance, and a higher rate of discontinuation and dose reduction.^24^ Rapamycin is another mTOR inhibitor that targets the PI3K/AKT/mTOR pathway and blocks downstream signaling. As an anti‐tumor treatment, rapamycin is usually highlighted for antitumor proliferation and anti‐tumorigenic properties and fewer adverse effects, better patient tolerance, and the potential to improve prognosis notably.^25^

Inetetamab (Septrin®), the first recombinant human monoclonal antibody developed and marketed in China, has the same two antigen binding fragments with 214 amino acids each, but with amino acid modifications to the fragment crystallizable region and manufacturing process optimization, exhibiting the same HER2 antigen binding affinity and greater antibody‐dependent cell‐mediated cytotoxicity as trastuzumab.^26^ Results from the Lan et al.^27^ had highlighted that inetetamab combined with pyrotinib and capecitabine showed complete tumor growth inhibition, and better antitumor activity than trastuzumab combined with pertuzumab plus capecitabine. In this study, the patient developed liver metastasis and was accompanied by PIK3CA gene mutation after trastuzumab treatment, treated with mTOR inhibitor in combination with the anti‐HER‐2 agent inetetamab. The choice of combination chemotherapy drugs has remained a tough problem in combination cancer therapy. In a study of patients with metastatic breast cancer, results suggested that the efficacy of albumin paclitaxel was not affected in patients previously treated with paclitaxel‐based drugs^28^ with higher efficacy.^29^ Therefore, the chemotherapeutic agent of choice was albumin paclitaxel monotherapy. Ultimately, the optimal regimen for patient was rapamycin combined with inetetamab combined with albumin paclitaxel monotherapy. The patient was well tolerated and had a progression‐free survival of 317 days.

The prospective, randomized controlled clinical trial of the patients enrolled in this case report aimed to explore the efficacy and safety of trastuzumab treatment in patients with metastatic HER‐2‐positive breast cancer who progressed after trastuzumab treatment with aberrant activation of the PI3K/AKT/mTOR pathway, to compare the efficacy and safety of inetetamab combined with rapamycin and chemotherapy regimens with that of standard treatment, and to explore new treatment options for trastuzumab‐resistant patients. Patients aged >18 years, diagnosed with HER‐2‐positive breast cancer, who experienced relapse after the completion of adjuvant trastuzumab therapy or PD during the course of adjuvant therapy, who experienced PD within ≥4 weeks after trastuzumab first‐line therapy, who had abnormal activation of PI3K/AKT/mTOR pathway, with an Eastern Cooperative Oncology Group score of ≤2, with clear measurable lesions, and without severe underlying cardiac, hepatic, or renal disease were included in the clinical trial. Patients who previously used pyrrolizidine and mTOR inhibitors and who developed symptomatic central nervous system metastases, gastrointestinal dysfunction, and chronic liver disease were excluded. The primary endpoint was PFS, while the secondary endpoints were objective response rate, overall survival, clinical benefit rate, and safety of patients. Six mutations in the PIK3CA gene were detected by the digital PCR method, including N345K, E726K, E542K, E545K, H1047L, and H1047R. If any of the above loci undergo mutations, we believe that the patient carries a PIK3CA gene mutation. The patient met the inclusion criteria (with no exclusions), signed the informed consent, and was assigned to the intervention group. The patient had normal tumor index test result prior to the study enrollment (Table 1), and tumor indexes were considered insensitive and were not used as a monitoring index.

**TABLE 1:** Tumor indicators prior to the study enrollment.

Index	Result	Unit	Reference values
Alpha‐fetoprotein (AFP1)	2.6	ng/mL	0 ~ 7
Carcinoembryonic antigen (CEA)	3.3	ng/mL	0 ~ 5
CA125	15.2	U/mL	0 ~ 25
CA19‐9	24.69	U/mL	0 ~ 30
CA15‐3	5.7	U/mL	0 ~ 24
Ferritin	115.9	μg/mL	13 ~ 150
CA724	1.77	IU/mL	0 ~ 8.2
Calcitoninogen	0.021	ng/mL	0 ~ 0.046

## 5 CONCLUSION

In this study, patient who progressed after trastuzumab treatment and had abnormal activation of the PI3K/Akt/mTOR pathway were selected to receive inetetamab combined with rapamycin to reverse drug resistance and restore the tumor sensitivity to anti‐HER2 monoclonal antibodies, achieving 317 days of PFS and tolerable adverse effects, and improving the prognosis. This was only a case study; a study on the efficacy and safety of inetetamab combined with rapamycin and chemotherapy in patients with metastatic HER2‐positive breast cancer with abnormal activation of the PI3K/AKT/MTOR pathway who have progressed after trastuzumab treatment is still ongoing. We look forward to the final results of this study, which will provide a new treatment option for patients with HER‐2‐positive PI3K mutations.

## AUTHOR CONTRIBUTIONS

All authors had full access to the data in the study and take responsibility for the integrity of the data and the accuracy of the data analysis. Conceptualization: Aijuan Wang and Chenghui Li. Methodology: Aijuan Wang and Chenghui Li. Investigation: Qi'an Jiang and Shu Jiang. Writing – original draft: Aijuan Wang. Writing – review and editing: Chenghui Li, Qi'an Jiang, and Shu Jiang.

## CONFLICT OF INTEREST STATEMENT

The authors have stated explicitly that there are no conflicts of interest in connection with this article.

## ETHICS STATEMENT

The clinical trial was approved by the hospital ethics committee (Medical Ethics [2021] No. 21), and informed consent was obtained from all study patients.

## INFORMED CONSENT

Informed consent was taken from the patient for the publication of case details and photographs.

## Data Availability

The data that support the findings of this study are available from the corresponding author.
